# Leveraging voice biomarkers to quantify chronic pain: a rapid review

**DOI:** 10.3389/fpain.2025.1678160

**Published:** 2026-01-12

**Authors:** Leah Tobey-Moore, Anu Iyer, Cade Wilkerson, Caraline Annichiarico

**Affiliations:** 1 Department of Psychiatry, Center for Health Services Research, College of Medicine, University of Arkansas for Medical Sciences, Little Rock, AR, United States; 2 Department of Geriatrics, College of Medicine, University of Arkansas for Medical Sciences, Little Rock, AR, United States; 3 Undergraduate School, Georgia Institute of Technology, Atlanta, GA, United States; 4 Medical School, College of Medicine, University of Arkansas for Medical Sciences, Little Rock, AR, United States; 5 Division of Academic Affairs, University of Arkansas for Medical Sciences, Little Rock, AR, United States

**Keywords:** artificial intelligence, chronic pain, digital health, machine learning, objective pain assessment, pain management, voice biomarkers

## Abstract

This rapid review examines the emerging role of voice-based artificial intelligence (AI) technologies in the objective assessment of chronic pain. It highlights promising applications of vocal biomarkers in pain quantification, particularly for populations with communication challenges or poorly understood conditions. AI is transforming healthcare, particularly for early detection of cardiovascular diseases and some neurological disorders, and it holds promise for managing chronic pain. This rapid literature review explores the potential of voice-based AI technologies to identify and analyze biomarkers that can objectively assess pain for populations with chronic pain conditions. These conditions are often complex and would benefit from more precise, reproducible measures of pain. While traditional pain scales heavily rely on self-reports, voice biomarkers are a non-invasive, scalable alternative. Studies show that changes in vocal characteristics—such as pitch, loudness, and jitter—correlate with pain intensity and quality; therefore, they offer insights that traditional, subjective measures may overlook. Machine learning models applied to voice data have demonstrated promise in detecting pain, particularly in vulnerable populations, such as those with intellectual and developmental disabilities. The review highlights how AI-driven voice analysis can complement cognitive behavioral therapy in pain management, enhancing accessibility and clinical outcomes. Despite the promise of AI-based approaches, challenges remain in standardizing these technologies for routine clinical use. Future research is needed to validate voice biomarkers across diverse pain conditions and to integrate them into clinical workflows to improve early diagnosis and personalized care, thus offering an innovative approach to chronic pain management. Key words: Voice biomarkers, Artificial intelligence, Pain Management, Machine Learning, Chronic Pain, Digital Health, Objective Pain Assessment

## Introduction

Artificial intelligence (AI) holds great promise for improving the objective assessment of chronic pain (1, 2), a complex and largely subjective condition that affects millions of adults worldwide (2, 3). The International Association for the Study of Pain (IASP) defines chronic pain as pain that persists or recurs for longer than three months, while acute pain typically has sudden onset and limited duration, usually less than a month (4). The causes of chronic pain are multifactorial and typically categorized as primary or secondary. Chronic primary causes refer to pain syndromes that cannot be explained by another diagnosis, such as fibromyalgia (4). Chronic secondary causes occur when pain arises from an identifiable structural pathology or underlying disease, such as post-surgical scar formation, trauma, or musculoskeletal pain (4, 5). Acute pain, on the other hand, usually results directly from surgery, illness, or trauma, and resolves as the underlying cause heals (6). Chronic musculoskeletal pain impacts more than 100 million adults (40%–60% of the U.S. population) annually, with higher prevalence among women and those with multiple chronic conditions (3, 7, 8). This results in an estimated $261–300 billion in annual healthcare expenses—more than heart disease, diabetes, and cancer combined (3). One of the most common and burdensome types of chronic pain is back pain, affecting up to 85% of adults during their lifetime (8, 9).

Persistent spinal pain syndrome (PSPS) represents a particularly challenging form of chronic low back pain, often arising after surgery or persisting despite standard treatment (10, 11). Its clinical presentation is highly variable, and symptoms are frequently underreported or difficult to quantify (10, 11). This variability highlights the limitations of current assessment practices and underscores the need for more objective approaches, particularly in conditions where pain severity and functional impact can be difficult to capture through traditional measures (1, 2, 10–13).

Traditional pain assessment tools, such as the visual analog scale (VAS) (14) and numeric rating scale (*N*RS) (15, 16), rely entirely on patient self-reporting. While commonly used, these tools provide a simplistic, one-dimensional snapshot of a singular point in time (17, 18). They do not account for the fluctuating and multidimensional nature of pain experiences and can frustrate patients who must assign a static number to a dynamic condition (10–12). These limitations also pose challenges for clinicians attempting to interpret scores accurately and develop appropriate treatment plans (10–12). Therefore, there is a critical need for more objective and consistent methods to assess pain severity, particularly for complex conditions like PSPS (10, 11). Traditional self-report tools remain essential but are constrained by subjectivity, recall bias, and variability across patients and clinical contexts (10–12).

Recent advances in AI technologies, including voice-based conversational agents and vocal biomarker analysis, have shown early promise in detecting and monitoring a range of medical conditions, including cardiovascular disease, Parkinson's disease, and mental health disorders (1, 19, 20). Vocal biomarkers, including patterns in pitch, tone, cadence, and articulation, represent scalable, noninvasive, and cost-effective indicators that may extend to pain assessment (1, 2, 13). Although applications of voice-based AI for pain quantification remain at an early stage, emerging work suggests that these approaches may complement traditional tools and provide a more nuanced understanding of patients’ pain experiences (2, 21, 22).

Other physiologic modalities such as functional near-infrared spectroscopy (fNIRS) (23) and electrocardiograms (EKG) (24) have likewise demonstrated potential in objectively characterizing pain by integrating hemodynamic or autonomic markers with machine learning (24, 25). These examples illustrate a broader shift toward objective, technology-supported pain evaluation; however, the present review focuses specifically on vocal-based approaches.

We conducted a rapid review of the literature to assess the current landscape of objective chronic pain evaluation, including AI technologies applied, reported findings, and patient populations studied. This review aims to identify gaps and opportunities in voice AI-driven pain assessment and to guide future research toward reliable, objective tools capable of improving chronic pain diagnosis, monitoring, and treatment.

## Methods

### Justification for rapid review approach

This review was conducted as a rapid review, following guidance from the Cochrane Rapid Reviews Methods Group (26), to provide a timely synthesis of a rapidly evolving field. AI, particularly voice-based technologies, has seen an exponential growth in healthcare applications over the past decade, especially in the development of diagnostic aids. These innovations are advancing more quickly than traditional systematic review timelines can accommodate (27, 28). The rapid review approach enabled the research team to balance methodological rigor with the need to synthesize current evidence in a timely manner, supporting both clinician decision-making and future research on AI-driven pain assessment.

### Literature search

A protocol was developed for internal documentation that outlined the review process and provided guidance and team expectations. A medical librarian developed a comprehensive search strategy in collaboration with the pilot study team, targeting peer-reviewed articles. This two-concept search strategy consisted of terminology for pain measurement and voice-based biomarkers. A third concept was added to help remove pediatric populations, animals, or non-human subjects. Advanced searching techniques included the use of controlled vocabulary, text words, truncation, nesting, and adjacency searching. A second medical librarian independently peer-reviewed the search strategy using the Peer Review of Electronic Search Strategies (PRESS) checklist to ensure completeness and precision (29). Electronic databases searched on March 29, 2024, were MEDLINE via OVID, Embase, and PsycINFO via EBSCO. The search terms and syntax were adjusted to fit each database. Database filters utilized were English language and publication dates between 2014 and 2024. Given the rapid review design and the need to capture recent advancements in AI, this 10-year window was selected to align with the surge in voice-based AI development and its application in health services and medical research. The final MEDLINE strategy is presented in Table 1; search strategies from the other databases are available upon request.

**Table 1:** Ovid MEDLINE search strategy.

Search #	Search terms	Results
1	exp Pain Measurement/ or (pain* adj2 (assess* or measur* or test* or scale* or questionnaire* or instrument* or tools* or evaluat* or investigat* or detect*)).mp.	146,683
2	(exp Visual Analog Scale/ or “visual analog scale”.mp.) and (exp Pain/ or pain.mp.)	28,448
3	1 or 2	158,797
4	exp Voice/ or ((voice or vocal*) adj2 (characteri* or analy* or qualit* or expression* or response* or biomarker*)).mp.	21,629
5	3 and 4	150
6	5 not (infant* or pediatric* or paediatric* or newborn* or animal* or veterinary* or mouse or mice or murine or mus or rat or rats or rattus or rodentia or hamster or guinea or rabbit* or pig or pigs or piglet* or porcine or swine or sow or sows or bird* or frog* or xenopus or cat or cats or feline* or canine* or dog or dogs or cow or cows or calve* or in or horse* or mares or foals or sheep or lamb* or ewes or ferret* or monkey* or mosquito* or simian* or baboon* or chimpanzee* or dolphin* or chick* or skink* or bats or woodchuck* or reptile* or lizard* or turtle* or zebrafish).mp.	96
7	limit 6 to (English language and yr=“2014–2024”)	57

### Study selection

The initial search yielded 141 articles, which were imported into Covidence, an online systematic review management platform. Covidence automatically identified and removed 17 duplicates, leaving 124 studies for screening. Three reviewers independently screened titles and abstracts using specified pre-defined exclusion criteria from the protocol: pediatric populations (<17 years), cancer-related pain, conference proceedings, surveys, non-human studies, and qualitative-only designs. Inclusion criteria consisted of studies examining musculoskeletal or chronic pain (e.g., back pain and complex regional pain syndrome) and those involving assessment tools (various scales, tests) related to pain measurement and voice biomarkers. Relevant preprints and abstracts were considered during screening, consistent with rapid review methodology.

**Figure 1:**
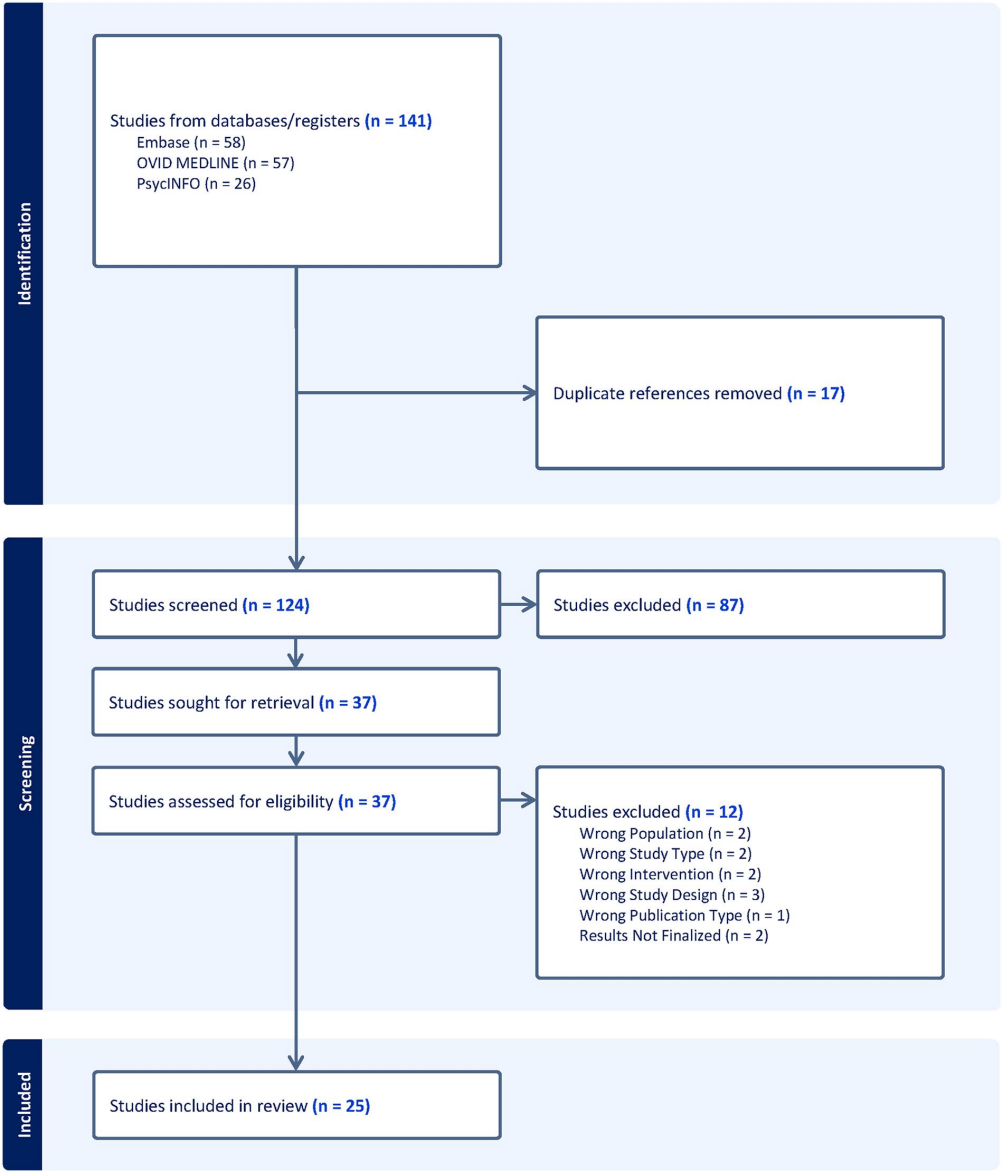
PRISMA (preferred reporting items for systematic reviews and meta-analyses) flow diagram generated using covidence systematic review software.

Each title and abstract were reviewed by two team members, with any conflicts adjudicated by a third reviewer of the research team. Eighty-seven irrelevant articles were excluded during the initial title and abstract screening. Full-text reviews were conducted on the remaining 37 articles using the same inclusion and exclusion criteria. Twelve studies were excluded after full-text review for reasons including ineligible population, study design, intervention, unfinalized results, and publication type (see Figure 1). Disagreements during full-text screening were resolved by consensus. Consistent with rapid review methodology (26–28), study selection was conducted using a dual-reviewer model to expedite the process with preserving rigor.

### Data extraction

Following study selection, reviewers developed a structured data extraction template within Covidence capturing key elements aligned with the research objective. Data fields included study design, population, characteristics, intervention type, outcomes related to voice and pain, and indicators relevant to risk of bias. The 25 included studies represented a range of study designs, including randomized controlled trials (RCTs), observational studies, systematic reviews, and pilot or feasibility trials. One reviewer conducted primary data extraction, and a second reviewer independently verified all entries to ensure completeness and accuracy.

### Risk of bias assessment

Given the heterogeneity in study designs and the emerging nature of the evidence base, we assessed risk of bias to enhance interpretability and rigor (27, 28), to identify high quality studies. Validated tools were applied to evaluate the quality of applicable studies. The standard Covidence quality assessment template based on the Cochrane Risk of Bias version 1 tool (RoB 1) was used for RCTs (30); the Risk Of Bias In Non-Randomized Studies-of Interventions tool (ROBINS-I) was used for non-randomized studies (31); and the AMSTAR 2 (A Measurement Tool to Assess Systematic Reviews) was used for systematic reviews (32). One reviewer completed the initial quality appraisal, and a second reviewer verified all judgments for accuracy and consistency.

## Results

### Study selection and characteristics

A total of 25 sources met inclusion criteria for this rapid review, including one systematic review (2), which was counted as a single evidence source to avoid duplication. These studies, summarized in Table 2, investigated voice-related features, machine learning models (MLMs), and digital tools to detect or quantify pain across a range of populations and study designs. Included study designs comprised RCTs (22, 33–37) such as those evaluating interactive voice response (IVR)-based cognitive behavioral therapy (CBT) for chronic back pain (22, 33); cohort and cross-sectional studies (38–43); feasibility and diagnostic accuracy studies (21, 44–46), and one systematic review (2). Populations studied included individuals with chronic musculoskeletal pain (42), voice disorders involving musculoskeletal symptoms (e.g., behavioral dysphonia) (38, 39, 47), intellectual and developmental disabilities (21), and substance use disorders (43), as well as healthy participants undergoing pain induction (48, 49). Others focused on vocally demanding occupations such as schoolteachers (33), musical theater actors (50), and tele-operators (40).

**Table 2:** Summary of studies included in the rapid review of AI-driven and voice-based methods for objective pain assessment.

Study ID	Design	Population	Intervention	Outcomes
Andriollo et al. 2023 (33)	Randomized controlled trial	Teachers with vocal and musculoskeletal complaints (*N* = 56)	Myofascial release with pompage	Improvement in musculoskeletal symptoms, quality of life, and social participation
Borna et al. 2023 (2)	Systematic review	Adults (*N* = 35)	Artificial Intelligence models for pain detection	Improved accuracy in analgesic prescription and patient assessment
Courbalay et al. 2018 (34)	Randomized controlled trial	Pain-experienced and non-specialist participants (*N* = 56)	Visual analog scale and gait analysis	Clinical experience influences pain assessment
D'haeseleer et al. 2022 (50)	Observational	Musical theater actors (*N* = 30)	Voice assessments via various questionnaires	Increased vulnerability to voice disorders
DosSantos et al. 2019 (40)	Cross-sectional	Tele-operators (*N* = 35)	Musculoskeletal pain and vocal fatigue questionnaires	Musculoskeletal pain increases vocal fatigue
Enguidanos et al. 2014 (44)	Feasibility study	Older adults (*N* = 27)	Smartphone pain application training	Interest in mobile pain monitoring with better training
Falbot et al. 2022 (41)	Cross-sectional	Dysphonic women (*N* = 30)	Pain and sleep quality assessment	Increased pain and sleep issues in dysphonic women
Ghai et al. 2014 (42)	Cohort	Chronic pain patients (*N* = 20)	Ultrasound-guided stellate ganglion block	Safe treatment for chronic pain
Gorfinkel et al. 2021 (43)	Cohort	Adults with substance use issues (*N* = 211)	Cannabis for opioid use	Cannabis not effective in reducing non-medical opioid use
Heapy et al. 2017 (22)	Randomized controlled trial	Chronic back pain patients (*N* = 125)	Interactive virtual reality cognitive behavioral therapy vs. in-person cognitive behavioral therapy	No significant difference in pain reduction
Heapy et al. 2016 (35)	Randomized controlled trial	Chronic low back pain patients (*N* = 230)	Interactive virtual reality-based cognitive behavioral therapy	Interactive virtual reality-based cognitive behavioral therapy as an alternative to in-person therapy
Heapy et al. 2014 (51)	Secondary data analysis	Chronic pain patients (*N* = 43)	Multiple pain ratings	Multiple-day reporting improves sensitivity in pain treatment trials
Icht et al. 2021 (21)	Diagnostic Test Accuracy Study	Adults with Intellectual and Developmental Disabilities (*N* = 30)	Pain vocalization recordings	Potential use of audio signals for pain monitoring in intellectual and developmental disabilities.
Keogh 2018 (48)	Experimental	Adults (*N* = 41)	Pain vocalization analysis	Task instructions affect sensitivity to pain vocalizations
Lautenbacher et al. 2017 (49)	Quasi-experimental	Students (*N* = 50)	Thermal pain stimulation and vocal analysis	Pain vocalizations affected pitch and loudness
Luyten 2016	Prevalence study	Flemish population (*N* = 333)	Voice therapy disorder, voice handicap index, and pain scales	High prevalence of voice therapy disorder, though severity is low
Mokhlesin et al. 2023 (36)	Randomized Controlled Trial	Patients with primary muscle tension dysphonia (*N* = 20)	Cricothyroid visor maneuver	Improved voice quality and reduced pain
Ramos et al. 2018 (47)	Case-control	Adults with dysphonia (*N* = 74)	Voice-related quality of life and musculoskeletal pain questionnaire	Dysphonic patients report higher pain intensity near larynx
Rankin 2019	Best-worst scaling experiment	Final year medical students (*N* = 237)	Chronic pain management factors	Highlights importance of chronic pain management education
Reimann et al. 2016 (38)	Cohort	Dysphonic individuals (*N* = 30)	Laryngeal manual therapy	Manual laryngeal therapy reduces pain in dysphonic patients
Ribeiro et al. 2019 (39)	Cohort	Women with behavioral dysphonia (*N* = 22)	Voice therapy program	Voice therapy program improves voice quality, symptoms, and musculoskeletal pain
Rose et al. 2015 (46)	Feasibility study	Primary Care Physician patients (*N* = 8,490)	Interactive Virtual Reality-based pre-screening for behavioral health	Validates Interactive Virtual Reality for pre-appointment screenings
Shahid et al. 2023 (45)	Diagnostic test accuracy study	Family caregivers (*N* = 30)	Critical pain observation tool for family caregivers	The critical pain observation tool for family caregivers improve pain detection in intensive care unit patients
Tohidast et al. 2022 (55)	Development and validation	Patients with voice disorders (*N* = 45)	Voice-related pain scale	Validates Voice-Related Pain Scale as a reliable pain assessment tool
Williams et al. 2014 (37)	Randomized controlled trial	Irritable Bowel Syndrome with Constipation patients (*N* = 1,608)	Irritable bowel syndrome with constipation symptom severity measures	Validates reliability of symptom severity measures in irritable bowel syndrome with constipation
Andriollo et al. 2023 (33)	Randomized controlled trial	Teachers with vocal and musculoskeletal complaints (*N* = 56)	Myofascial release with pompage	Improvement in musculoskeletal symptoms, quality of life, and social participation
Borna 2023 (2)	Systematic review	Adults (*N* = 35)	Artificial Intelligence models for pain detection	Improved accuracy in analgesic prescription and patient assessment
Mokhlesin et al. 2023 (36)	Randomized controlled trial	Patients with primary muscle tension dysphonia (*N* = 20)	Cricothyroid visor maneuver	Improved voice quality and reduced pain
Shahid et al. 2023 (45)	Diagnostic test accuracy study	Family caregivers (*N* = 30)	Critical Pain Observation Tool for Family caregivers	The critical pain observation tool for family caregivers improve pain detection in intensive care unit patients
Tohidast et al. 2022 (55)	Development and validation	Patients with voice disorders (*N* = 45)	Voice-related pain scale	Validates voice-related pain scale as a reliable pain assessment tool
D'haeseleer et al. 2022 (50)	Observational	Musical theater actors (*N* = 30)	Voice assessments via various questionnaires	Increased vulnerability to voice disorders
Falbot et al. 2022 (41)	Cross-sectional	Dysphonic women (*N* = 30)	Pain and sleep quality assessment	Increased pain and sleep issues in dysphonic women
Gorfinkel et al. 2021 (43)	Cohort	Adults with substance use issues (*N* = 211)	Cannabis for opioid use	Cannabis not effective in reducing non-medical opioid use
Icht et al. 2021 (21)	Diagnostic test accuracy study	Adults with intellectual and developmental disabilities (*N* = 30)	Pain vocalization recordings	Potential use of audio signals for pain monitoring in intellectual and developmental disabilities.
DosSantos et al. 2019 (40)	Cross-sectional	Tele-operators (*N* = 35)	Musculoskeletal pain and vocal fatigue questionnaires	Musculoskeletal pain increases vocal fatigue
Rankin 2019	Best-worst scaling experiment	Final year medical students (*N* = 237)	Chronic pain management factors	Highlights importance of chronic pain management education
Ribeiro et al. 2019 (39)	Cohort	Women with behavioral dysphonia (*N* = 22)	Voice therapy program	Voice Therapy Program improves voice quality, symptoms, and musculoskeletal pain
Courbalay et al. 2018 (34)	Randomized controlled trial	Pain-experienced and non-specialist participants (*N* = 56)	Visual analog scale and gait analysis	Clinical experience influences pain assessment
Keogh 2018 (48)	Experimental	Adults (*N* = 41)	Pain vocalization analysis	Task instructions affect sensitivity to pain vocalizations
Ramos et al. 2018 (47)	Case-control	Adults with dysphonia (*N* = 74)	Voice-related quality of life and musculoskeletal pain questionnaire	Dysphonic patients report higher pain intensity near larynx
Lautenbacher et al. 2017 (49)	Quasi-experimental	Students (*N* = 50)	Thermal pain stimulation and vocal analysis	Pain vocalizations affected pitch and loudness
Heapy 2017 (22)	Randomized controlled trial	Chronic back pain patients (*N* = 125)	Interactive virtual reality cognitive behavioral therapy vs. in-person cognitive behavioral therapy	No significant difference in pain reduction
Heapy et al. 2016 (35)	Randomized controlled trial	Chronic low back pain patients (*N* = 230)	Interactive virtual reality-based cognitive behavioral therapy	Interactive virtual reality-based cognitive Behavioral Therapy as an alternative to in-person therapy
Luyten 2016	Prevalence study	Flemish population (*N* = 333)	Voice therapy disorder, voice handicap index, and pain scales	High prevalence of voice therapy disorder, though severity is low
Reimann et al. 2016 (38)	Cohort	Dysphonic individuals (*N* = 30)	Laryngeal manual therapy	Manual laryngeal therapy reduces pain in dysphonic patients
Rose et al. 2015 (46)	Feasibility study	Primary care physician patients (*N* = 8490)	Interactive virtual reality-based pre-screening for behavioral health	Validates interactive virtual reality for pre-appointment screenings
Enguidanos et al. 2014 (44)	Feasibility study	Older adults (*N* = 27)	Smartphone pain application training	Interest in mobile pain monitoring with better training
Ghai 2014 (42)	Cohort	Chronic pain patients (*N* = 20)	Ultrasound-guided stellate ganglion block	Safe treatment for chronic pain
Heapy et al. 2014 (51)	Secondary data analysis	Chronic pain patients (*N* = 43)	Multiple pain ratings	Multiple-day reporting improves sensitivity in pain treatment trials
Williams et al. 2014 (37)	Randomized controlled trial	Irritable bowel syndrome with constipation patients (*N* = 1608)	Irritable bowel syndrome with constipation symptom severity measures	Validates reliability of symptom severity measures in irritable bowel syndrome with constipation

Pain was primarily assessed using self-report instruments, including the NRS (22, 35, 37, 49, 51), VAS (21, 34), and the musculoskeletal pain questionnaire (38–41, 47). Several studies incorporated acoustic, perceptual, or prosodic analysis of vocal data, either to support pain detection (21, 48) or to assess outcomes of voice-focused interventions (36, 38, 39). The systematic review examined how MLMs could classify pain states based on vocal features and integrate voice into multimodal pain monitoring frameworks (2).

### Risk of bias summary

**Figure 2:**
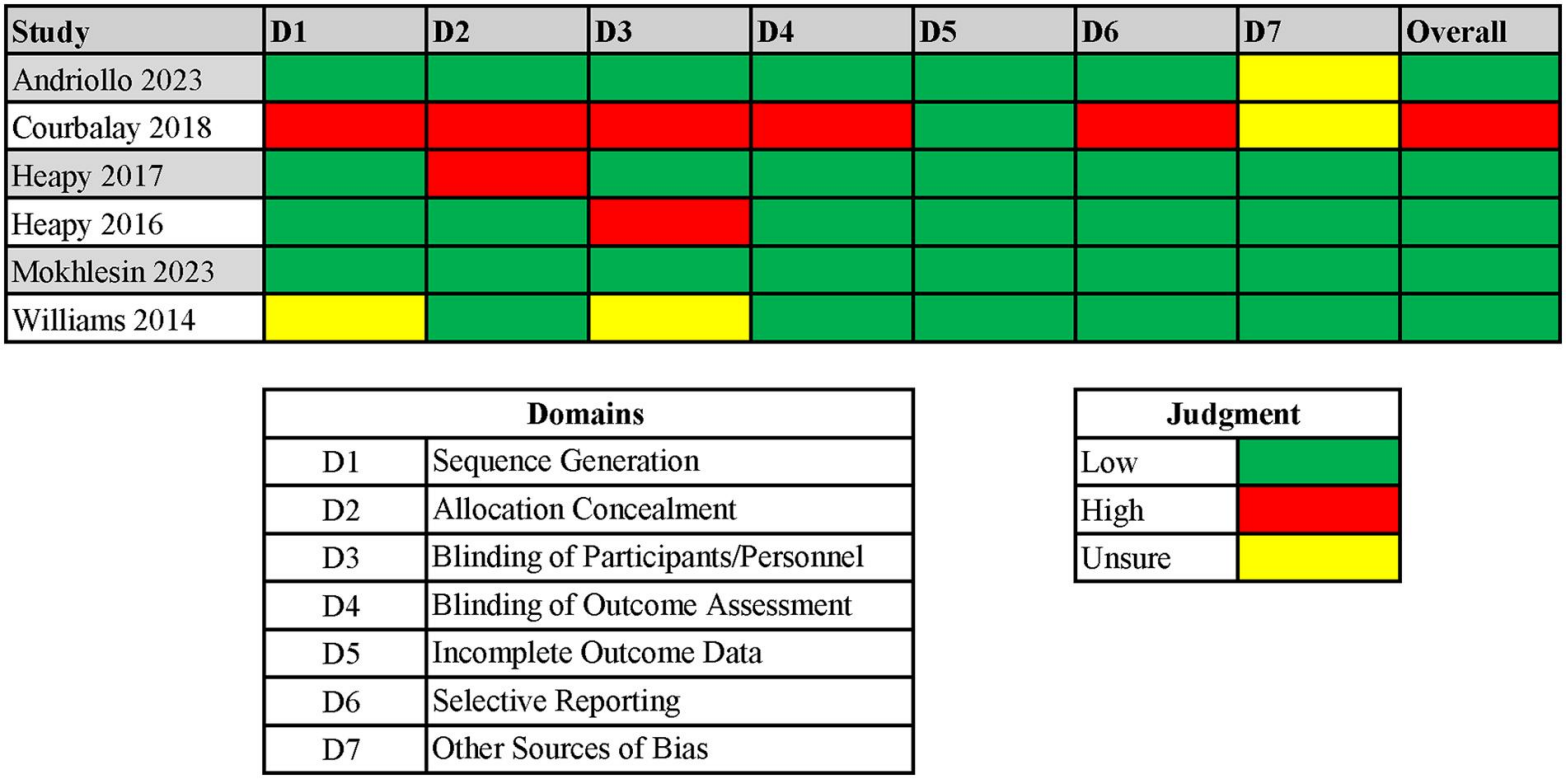
Rob 1 (risk of bias version 1 tool). Study-level assessments are presented across seven Cochrane Risk of Bias domains: sequence generation (D1); allocation concealment (D2); blinding of participants and personnel (D3); blinding of outcome assessment (D4); incomplete outcome data (D5); selective reporting (D6); and other sources of bias (D7). Using Cochrane guidance, the reviewers rated each domain as Low, High, or Unclear risk of bias. The overall risk for each study was evaluated using the individual results from each domain.

**Figure 3:**
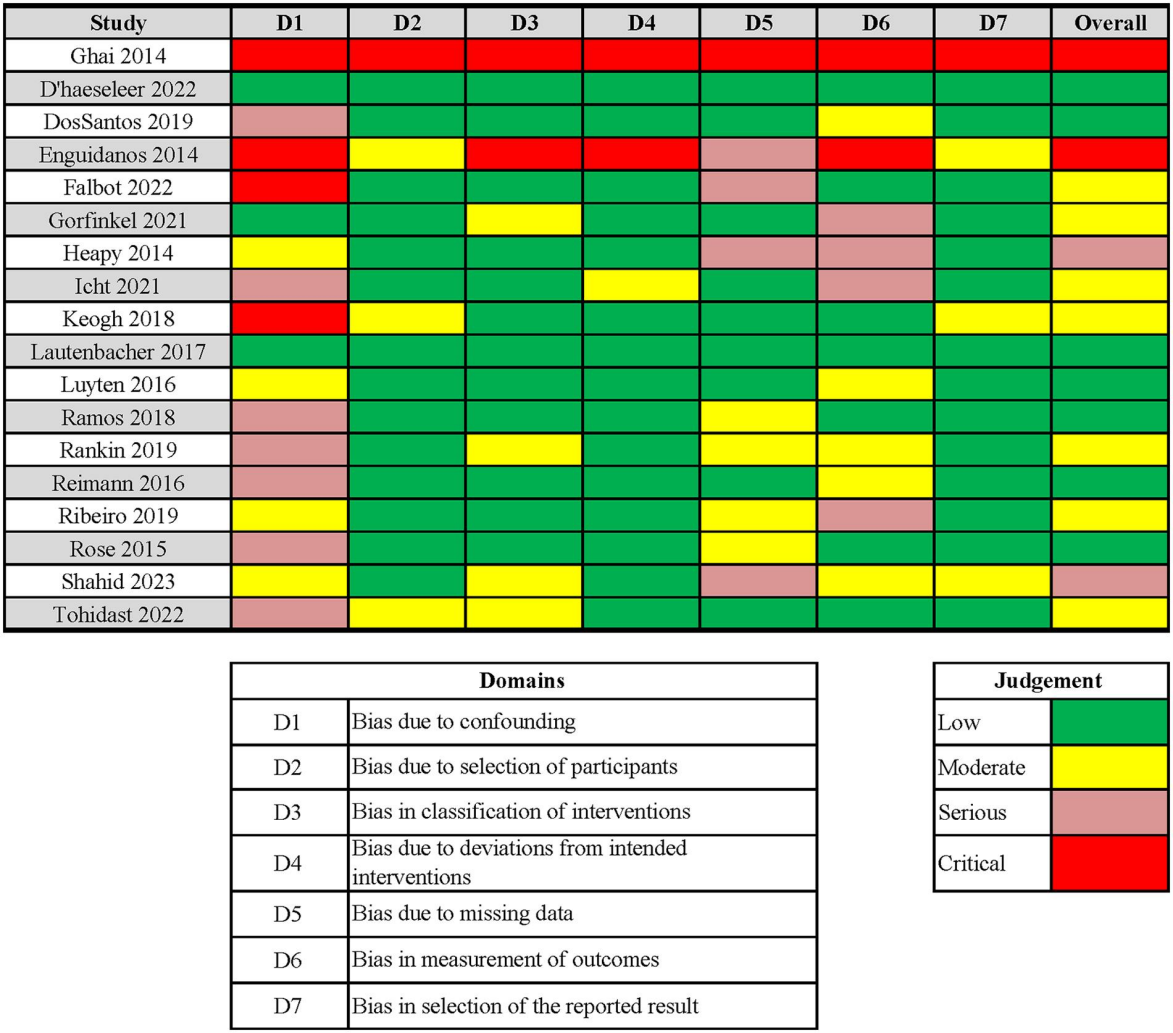
ROBINS-I (risk of bias in Non-randomized studies—of interventions). Study-level judgments are shown across seven ROBINS-I domains: bias due to confounding (D1); bias in selection of participants (D2); bias in classification of interventions/exposures (D3); bias due to deviations from intended interventions (D4); bias due to missing data (D5); bias in measurement of outcomes (D6); and bias in selection of the reported result (D7). Reviewers used standard ROBINS-I categories (Low, Moderate, Serious, Critical) to assess the overall risk for each study.

Included studies were assessed using risk of bias tools appropriate to their design. RCTs, evaluated using the RoB 1 tool, were rated as low to moderate risk. Non-randomized studies of interventions, assessed using the ROBINS-I tool, were rated as moderate to serious risk. The single systematic review (2) was appraised using AMSTAR 2 and received a rating of low confidence, reflecting methodological limitations. Detailed results of risk of bias assessments are presented in Figures 2, 3.

### Voice biomarkers for pain detection

Several studies explored whether voice features could serve as objective indicators of pain intensity or experience. These studies typically examined acoustic characteristics such as pitch, jitter, loudness, harmonic-to-noise ratio, and vocalization duration, often using analytic tools like *Praat* software (48, 49). Other software such as OpenSMILE have been incorporated to extract many low-level descriptors that span spectral (e.g., harmonic-to-noise ratio), temporal (e.g., speech rate, pause distribution) and prosodic features (e.g., rhythm, intensity) features. These multidimensional features can capture subtle changes linked to pain as they provide a comprehensive representation of vocal function.

In a diagnostic test accuracy study, researchers analyzed audio recordings from individuals with intellectual and developmental disabilities and found that pain-related vocalizations exhibited longer durations and increased jitter (21). Although these features did not reach statistical significance, they highlight the potential for using voice analysis in populations with limited verbal communication. Performance metrics in these studies were reported with accuracy, sensitivity/specificity, or area under the curve (AUC). Accuracies ranged broadly (often between 60% and 85%), which underscores the need for reporting standardized metrics for cross-study comparison. Two experimental studies examined vocal responses to thermal pain (48, 49). Lautenbacher et al. reported that pitch and loudness increased during painful stimuli, especially during sustained vowels sounds such as/u/ and/ǝ/ (schwa), which also correlated with higher self-reported pain scores. Keogh et al. found that vocalizations were more influenced by task instructions (e.g., exaggerating vs. suppressing pain) than by pain intensity itself, raising questions about the contextual sensitivity of voice-based measures.

Only systematic review applied MLMs to voice data for pain detection. It concluded that AI models, when paired with audio-video recordings and clinical metadata, could enhance pain assessment precision compared to traditional self-report instruments (2). Across literature, Support Vector Machines (SVMs) and Random Forest classifiers have been previously applied to pain-related vocal features—and deep learning approaches with Convolutional Neural Networks (CNNs) and Recurrent Neural Networks (RNNs/LSTMs) have also demonstrated potential in speech modeling. However, in pain research, the combination of heterogeneous data (acoustic features and linguistic content) and limited sample sizes further constrains the application of deep learning models. Across studies, acoustic features such as pitch, jitter, and vocalization duration were frequently examined in relation to pain, though findings varied and were limited by small samples sizes and inconsistent methodologies.

### Voice changes in acute vs. chronic pain

Voice-related responses to acute and chronic pain were examined across several experimental and clinical contexts. Experimental studies used simulated pain models to evaluate vocal responses under tightly controlled conditions, offering insight into how acute pain may influence real-time vocal expression. Acute pain studies typically report increases in pitch, loudness, jitter, and speech rate—acoustic markers that reflect short-term changes. These studies also highlighted how behavioral factors, such as task instructions, can shape vocal output during painful stimuli (48, 49) (see *Voice Biomarkers for Pain Detection* subsection).

In contrast, studies focused on chronic pain primarily involved individuals with long-term vocal disorders and co-occurring musculoskeletal symptoms. Chronic pain-related voice changes tend to be reflected in sustained alternations in vocal quality and timing, such as lower intensity and reduced speech rate which can represent fatigue or adaptation to persistent pain. For example, several studies examined participants experiencing vocal strain, laryngeal discomfort, and neck or back pain alongside reduced voice quality (38, 39, 47). These findings suggest that chronic pain may affect both voice production and overall vocal function, with potential consequences for daily communication and quality of life. In a series of RCTs, CBT interventions were evaluated for patients with chronic low back pain, although vocal parameters were not explicitly analyzed as outcomes (22, 35, 51). Across studies, acute pain was typically investigated using brief, stimulus-driven tasks, whereas chronic pain research emphasized broader symptom profiles, including long-standing voice-related impairments, fatigue, and functional limitations.

### Pain voice datasets and annotation practices

Current voice datasets for pain detection remain limited in size and standardization. Most are derived from smaller studies under 100 participants, with annotations based on either self-reported pain intensity ratings or physiological factors. Furthermore, the lack of demographic diversity in addition to non-standardizable annotation practices complicates comparison across studies, causes reduced generalizability, and detrimentally impacts model training. Currently, there is no publicly available large-scale multimodal voice dataset specifically designated for pain assessment. The absence of consistent annotations and standardized metadata limits reproducibility across models. This gap underscores the need for developing a robust well-annotated dataset that captures acute and chronic pain expressions for generalizability and clinical application.

### Speech, pain, and quality of life

Several studies examined the downstream impact of pain and vocal dysfunction on daily functioning, communication, and psychosocial well-being. In an RCT, researchers found that myofascial release therapy improved musculoskeletal pain symptoms, enhanced quality of life, and supported greater social engagement among teachers with chronic voice complaints (33). Another study reported that higher levels of musculoskeletal pain were associated with increased vocal fatigue in tele-operators, a population at risk due to high vocal demands and repetitive strain (40).

Voice-related quality of life also emerged as a key concern in studies involving individuals with dysphonia. One study found that dysphonic adults reported higher pain intensity in the laryngeal region and scored lower on voice-related quality of life assessments compared to healthy controls (47). A separate study observed that a targeted voice therapy program led to improvements in vocal function and reductions in musculoskeletal pain and disability (39). These findings emphasize how chronic pain and voice dysfunction can co-occur and negatively affect daily activities, communication, and psychosocial well-being, particularly in vocally intensive or occupationally demanding contexts.

## Discussion

### Clinical and measurement challenges in chronic pain

Despite decades of research, chronic pain remains underdiagnosed, undertreated, and economically devastating, costing the U.S. over $500 billion annually in direct and indirect costs (3). Conditions such as chronic low back pain and persistent spinal pain syndrome (PSPS) presenting particularly complex challenges for diagnosis and treatment (10). These disorders are often accompanied by significant quality of life impairments, reduced productivity, and physically debilitating functional limitations (3, 8, 9).

Pain assessment remains challenging due to its subjective nature and the limitations of self-report tools like the NRS and VAS, which lack contextual sensitivity and may be interpreted differently depending on individual or situational factors (15). These tools also fail to capture important nuances, including functional interference and psychosocial factors shaping the pain experience (52). They also overlook population-level differences such as those related to age (16), and fluctuations in pain over time due to activity or emotional distress (15), further complicating accurate and consistent evaluation (52).

In contrast, some studies included in this review begin addressing these limitations by capturing task-or context-sensitive vocal responses (49) and daily fluctuations in pain using digital tools like IVR systems (22). These findings underscore the need for complementary, voice-based methods of pain assessment that extend beyond traditional self-report and can capture physiological or behavioral indicators of distress, especially in populations where verbal communication is impaired or unreliable (15, 16). Voice-based approaches offer a promising direction, as acoustic features such as pitch, loudness, jitter, and vocalization duration may reflect both the sensory and affective dimensions of pain (2, 48, 49).

Physiological AI modalities provide direct measures of nociceptive activity such as EEG (electroencephalogram) and EKG, but the primary advantages of voice-based AI is that it is noninvasive, low-cost and scalable to telehealth settings. In this work, we focus on a single-modality approach with voice data to detect pain. Future multimodal integration (such as combining voice with physiological signals) could provide a more accurate view of pain expression.

Despite these promising directions, many included studies relied on small, homogeneous samples or highly controlled laboratory settings, limiting generalizability to broader patient populations (41, 48, 49). Several studies used healthy participants in simulated pain conditions, while others focused on individuals with distinct occupational or vocal demands, such as teachers, tele-operators, or actors (33, 40, 50). Although some studies did include individuals with chronic musculoskeletal pain (22, 35, 40–42, 47), fewer examined diverse, medically complex or underrepresented populations, such as older adults (44) or individuals with communication impairments (38–40).

### Emerging role of voice-based AI in pain assessment

Voice-based tools varied widely in their design, including differences in recording conditions, selected acoustic features, and analytic methods. These inconsistencies limit cross-study comparability and hinder the development of standardized protocols. For example, some studies relied on sustained vowel production (e.g.,/u/,/ə/) under controlled conditions, while others analyzed spontaneous speech during social interaction or behavioral tasks (41, 49). The absence of consensus on clinically relevant vocal markers, combined with a lack of agreed-upon thresholds for identifying pain-related changes, poses a key barrier to clinical translation (2, 49).

Traditional self-report measures remain foundational in clinical practice, but their limitations are especially problematic for populations with communication barriers, such as individuals with intellectual and developmental disabilities or neurological conditions. In these groups, verbal reporting may be unreliable or impossible, creating an urgent need for complementary, objective tools to assess pain (18). Most standardized scales were developed for general adult populations and may not translate effectively across diverse clinical or cultural contexts (15, 16).

Voice-based approaches offer potential advantages in addressing these gaps (2). Acoustic characteristics such as pitch, loudness, jitter, and prosodic variation may reflect physiological or emotional responses to pain, making them promising noninvasive biomarkers of pain-related distress (2, 48, 49). These features have been shown to fluctuate during painful stimuli in individuals with chronic pain (41, 48, 49). Tools such as acoustic analysis software (e.g., Praat) and IVR systems have enabled vocal signal tracking over time, supporting their feasibility for longitudinal pain monitoring outside traditional clinical settings (2, 22). Such methods may be especially valuable in populations where standard assessments are unreliable or inaccessible (16, 21).

Experimental research underscores the potential of vocal features as dynamic indicators of acute pain, particularly in controlled settings (21, 48, 49). Rather than simply documenting vocal shifts during painful stimuli, these studies highlight the role of cognitive and contextual factors in shaping pain-related vocalizations. For example, one study showed that even basic acoustic parameters like pitch and loudness changed in response to thermal pain simulation (49), while another found that pain induced by a cold pressor task was associated with increased pitch and speech rate (21). In a separate study, participants instructed to exaggerate or suppress their pain significantly altered their vocal expression, raising concerns about the contextual reliability of voice-based pain indicators in clinical practice (48).

While much of this evidence centers on acute, stimulus-driven pain models, a smaller set of studies explored voice changes in chronic pain contexts. For example, one study reported that women with dysphonia and chronic musculoskeletal pain exhibited altered voice quality and reduced phonation stability, suggesting long-term vocal adaptations linked to persistent pain (41). These studies offer preliminary insights into how chronic pain may shape vocal function, especially in populations with musculoskeletal voice disorders or high vocal demands. Recent work has explored the potential of multimodal MLMs to improve the precision of pain detection by integrating vocal features with audio–video recordings and clinical metadata (2).

However, only one study from our search terms applied MLMs specifically to voice-based pain detection, highlighting a critical gap in the current evidence base (2). In related domains, MLMs have demonstrated high accuracy distinguishing clinical conditions such as Parkinson's disease using vocal input (20), further supporting the feasibility of these approaches. Further AI tools that combine subjective reports with objective vocal indicators may enhance real-time monitoring and support clinician decision-making, particularly in populations with communication impairments or fluctuating symptoms.

This review synthesized findings from 25 studies investigating voice as a tool for detecting or quantifying pain. Many of the studies analyzed acoustic features such as pitch, jitter, loudness, and speech rate, often using tools such as *Praat* software or machine learning classifiers to detect pain-related changes in vocalizations (2, 20, 22, 49). These vocal indicators were studied in both acute and chronic pain contexts, with evidence supporting measurable alterations in vocal output during painful experiences. For example, one study found that pitch and loudness varied significantly during painful thermal stimulation, particularly during sustained vowel production (e.g.,/ah/), suggesting that painful stimuli can induce consistent and quantifiable changes in vocal expression (49). If applied in chronic pain populations, spectrographic analysis, which functions like a vocal fingerprint visualizing vocal frequency and amplitude, may help identify subtle shifts in vocal effort related to breath control, muscular tension, or emotional stress (2, 20, 49).

Although our search strategy emphasized vocal characteristics and did not yield further studies on signal analysis or facial expressions, there is growing evidence supporting their utility in pain detection. For instance, recent work has advanced multimodal physiological signal analysis to more sensitively detect pain states beyond subjective report (53). In parallel, facial-expression-based methods using MLM have shown promise in inferring pain intensity from subtle facial movements (54). These findings point toward complementary modalities deserving future integration into rapid review frameworks.

### Intervention opportunities and multimodal integration

Several studies in this review explored how targeted interventions influenced both pain and vocal function. One RCT investigated myofascial release with pompage in teachers experiencing musculoskeletal and vocal complaints. Participants reported improvements in musculoskeletal symptoms, quality of life, and social participation, suggesting therapeutic benefits across both pain and voice-related outcomes (33).

Voice therapy techniques were also found to influence both vocal function and pain outcomes. For example, a pilot randomized trail tested the cricothyroid visor maneuver in individuals with primary muscle tension dysphonia, resulting in improvements in maximum phonation time and Consensus Auditory-Perceptual Evaluation of Voice (CAPE-V) scores, along with reductions in self-reported pain (36). Similarly, observational studies have shown that individuals with dysphonia often report greater musculoskeletal pain in the laryngeal and upper body regions compared to vocally healthy controls. Treatment with voice therapy in these populations was associated with improvements in both voice quality and musculoskeletal discomfort (38, 39).

Digital interventions were also prominent in the reviewed studies. An IVR system delivering CBT individuals with chronic low back pain was shown to be as effective as in-person CBT for reducing pain intensity and improving coping strategies (22). This indicates that voice-enabled platforms may support scalable, remote access to evidence-based interventions.

Some studies examined interventions beyond voice or behavioral therapy. For example, an ultrasound-guided stellate ganglion block was reported as a safe and effective treatment for patients with chronic sympathetic pain, emphasizing the importance of combining procedural techniques with accurate pain assessment tools (42). This reflects a broader trend towards integrating pharmacologic or interventional treatments with complementary assessment strategies to guide individualized pain management.

In addition to therapeutic outcomes, several studies highlighted occupational vulnerabilities to vocal and musculoskeletal complaints. For example, observational studies involving musical theater students and dysphonic women showed elevated levels of pain, sleep disturbance, and vocal fatigue in these populations, often linked to stress and repetitive voice use (41, 50). The co-occurrence of symptoms in these populations suggests that integrated care strategies targeting vocal and musculoskeletal demands may be warranted. A separate cohort study found that cannabis use was not effective in reducing nonmedical opioid use, emphasizing the need for evidence-based, multimodal strategies to address chronic pain management (43).

### Challenges and future directions

This review is limited by having only three databases searched that were highly relevant to the topic of voice vocalization characteristics in adults with reported pain. Date restrictions were limited to the past 10 years to review the most current literature relevant to new AI technology. Data collection was conducted in an expedited manner; where a single reviewer performed data extraction and assessed risk of bias, then a second reviewer checked and verified the data.

Several studies identified important challenges related to the feasibility, accessibility, and scalability of voice-and technology-based pain assessment tools. For example, one study found that older adults expressed interest in smartphone-based pain monitoring, but required additional support to use the application effectively, highlighting the need for user-friendly design tailored to this population (44). Another study validated the use of an IVR platform for pre-screening behavioral health symptoms in primary care (46), while a separate study demonstrated that a pain observation tool could be reliably administered by family caregivers in critical care settings when appropriate training and support were provided (45).

The included systematic review emphasized both the promise and limitations of AI-based models in pain detection (2). While these tools may improve diagnostic precision when paired with clinical data and voice features, important concerns remain. Key issues include the limited generalizability of current models beyond controlled settings (48, 49), a lack of standardization in voice data collection and acoustic feature extraction (41, 49), and the interpretability of AI-generated outputs (2). Relatively few studies evaluated how these tools can be integrated into routine clinical care, such as within electronic health records or telemedicine platforms, or assessed their usability among different populations (21, 45, 46).

Future research should prioritize large-scale validation studies in demographically and clinically diverse populations, including individuals with communication impairments, multiple chronic conditions, or limited access to specialty care (16, 21, 45). Interdisciplinary collaborations will be essential to advancing this work, bringing together clinicians, data scientists, speech-language pathologists, physical therapists, translational and implementation experts. Consensus on relevant vocal features, standardized recording protocols, and validated outcome measures will be critical to ensure reproducibility and clinical applicability (2, 49).

Finally, the development and validation of structured clinical instruments such as the Voice-Related Pain Scale (VRPS) (55), Voice Problem Impact Scale (VPIS) (56), and the Critical Pain Observation Tool for Family Caregivers (CPOT-Fam) (57), represent important steps toward bridging subjective and objective pain assessment (2, 21, 45). When combined with voice-based technologies, these tools may help providers and clinicians better identify pain-related vocal changes, especially in populations with limited ability to self-report. Future translational and implementation research should explore how best to integrate these tools into routine practice while ensuring accessibility, training support, and cultural and linguistic appropriateness.

## Conclusions

This review highlights the emerging role of voice-based AI in the assessment of pain across acute and chronic contexts. Across study designs and populations, findings support the potential of vocal biomarkers, such as pitch, loudness, jitter, and speech rate, as objective indicators of pain experience. These technologies may be especially beneficial in clinical contexts where traditional, subjective pain assessment methods are limited, including among individuals with communication barriers, fluctuating pain patterns, or multimorbidity.

Advancements in AI are enabling more scalable, noninvasive, and rapid approaches to pain detection. Recent work using spectrogram analysis and acoustic feature extraction demonstrates how voice can serve as a physiological signal of pain, providing additional insight beyond patient self-report. These tools hold particular promise for telemedicine and other settings where conventional assessment methods may be impractical or inconsistent.

Despite these advances, more research is needed to standardize voice data collection and analysis, validate models in diverse clinical populations, and integrate these tools into routine care. With continued interdisciplinary collaboration and a focus on equity and usability, voice-based AI technologies may ultimately help close the gap between subjective reports and objective measurement in chronic pain care.
